# River suspended sediment response to tropical cyclone precipitation in the Eastern United States

**DOI:** 10.21203/rs.3.rs-10373444/v1

**Published:** 2026-08-03

**Authors:** Luísa V. Lucchese, John R. Gardner, Hunter Quintal, Colin Gleason, Antonia Sebastian

**Affiliations:** 1 Department of Geology and Environmental Science, University of Pittsburgh, PA 15260-3332, United States of America; 2 Department of Earth, Marine and Environmental Sciences, University of North Carolina at Chapel Hill, NC 27599, United States of America; 3 Civil and Environmental Engineering, UMass Amherst, Amherst MA 01003, United States of America

## Abstract

We leverage remotely sensed observations of suspended sediment concentration and 4km hourly precipitation observations to analyze the effect of tropical cyclone (TC) precipitation on riverine suspended sediment flux in the eastern United States. We estimate 278,500 tons of sediment were transported annually due to TCs, approximately 3% of all sediment transported (0-22% depending on location). TC precipitation transports more sediment than comparable precipitation totals attributed to non-TC events, even in cases with lower discharge. We show that TC-related sediment load is independent from upstream reservoir volume, which normally down-regulates sediment flux. Further, TCs cause rapid increases in sediment transport over a precipitation threshold, unlike comparable non-TC precipitation. Previously only observed at the catchment scale, we provide the first large-scale evidence that TC and non-TC precipitation elicit fundamentally different river sediment responses. Differences in sediment responses between TC and non-TC precipitation can inform infrastructure planning and mitigation of water quality impacts.

## Introduction

Tropical cyclones (TCs) are rotating storm systems that can be classified as depressions, tropical storms, or hurricanes. TC rainfall can mobilize significant amounts of sediment into rivers via landscape erosion, bed and bank erosion, and mass wasting events such as landslides^1-5^. Consequences of TC-driven sediment flux include rapid reservoir infilling^6^ and costly drinking water treatment from riverine source waters^7^. While TC-driven erosion and sediment flux through rivers can be a destructive force, it accounts for a majority (30-90%) of the sediment that deltas and coastal wetlands need to maintain elevation in some locations^3,8^. The consequences of TC precipitation and the riverine sediment responses associated with them deserve attention through broader observational studies.

Past work suggests rivers may respond differently to TCs and non-TCs. In a recent work using hydrologic models, it was estimated that TCs transported six times more sediment compared to non-TC in a catchment in Fiji^9^. A TC triggered the highest ever recorded sediment concentration in a catchment in Taiwan^1^. The fraction of sediment flux that can be attributed to TCs was estimated at 12 and 21% for two rivers in China (rivers Nanliu and Qin)^10^. However, we have limited understanding of how riverine sediment flux responds to TC and non-TC events at large scales, and especially in the United States. To better constrain the role of TCs on riverine sediment flux to coasts, we need to directly link the spatial-temporal footprint of TC precipitation to riverine sediment fluxes using observations across a diverse set of watersheds, at the regional scale.

It is critical to understand the impact of TCs on sediment flux along the US east and Gulf coasts. The US east and Gulf coasts are a hotspot for TC activity as well as key deltas and coastal wetlands that are at risk of drowning^11^. TCs also drive significant coastal and inland flooding^12,13^ and alter carbon, nutrient and phytoplankton dynamics in rivers and estuaries across the eastern US^14^. Further, recent research suggests that TC tracks on the US east coast could be progressively migrating inland and northward^15^ (Fig. S01), increasing exposure of the continental US to TC precipitation-driven hazards^16^, as well as mobilizing sediment over larger contributing areas. Given climatic and hydrologic non-stationarity, observations of spatial-temporal precipitation and time series of sediment flux across many watersheds can help identify where TCs play an outsized role on sediment flux, potentially informing coastal conservation and disaster mitigation efforts.

However, measurements of riverine SSC and sediment flux during large events are rare. It is dangerous to conduct field campaigns during high flow events, and in-situ sensors can reach their maximum turbidity response^17^ or be damaged, buried, or washed out. Satellite remote sensing is a potential solution with a different set of limitations, such as sensor saturation at extreme SSC values^18^ and cloud cover^19^. However, satellites offer a safe and non-contact method for estimating SSC before, during, and right after TC and non-TC events, particularly in large rivers where hydrologic response is slower and the watershed outlet may not be exposed to the same cloud cover, providing windows of opportunity to capture TC events. Specifically, the Harmonized Landsat Sentinel-2 (HLS) product provides the most frequent (1.4 days on average) observations with a spatial resolution (30 m) that can resolve rivers that are > 50 m wide, and is a proven product for estimating SSC^20,21^.

Our goal is to quantify the fraction of sediment flux that is TC-driven and compare river sediment and discharge responses between TC and non-TC precipitation across 40 large rivers draining into the US East and Gulf coasts (Fig. S02, Fig. S03). We thus: 1) perform unsupervised clustering identifying TC and non-TC precipitation events and calculate their spatial and temporal footprint, and 2) execute a deep learning algorithm to estimate SSC from the Harmonized Landsat Sentinel-2 (HLS) satellite product. We focus on the period from 2013 to 2024, as the HLS record starts in 2013, and combine satellite-derived SSC with daily United States Geological Survey (USGS) discharge observations to estimate sediment flux. We recognize satellites capture SSC in the upper water column; however, our focus is on the relative differences in sediment flux across rivers and between TC and non-TCs, not on estimating the absolute total, depth-integrated sediment flux. Remote sensing is currently the only approach that can provide a consistent historical time series across many rivers and serves as an appropriate SSC estimate for our goals.

## Results

### TC response across the US east coast

**Figure 1 -:**
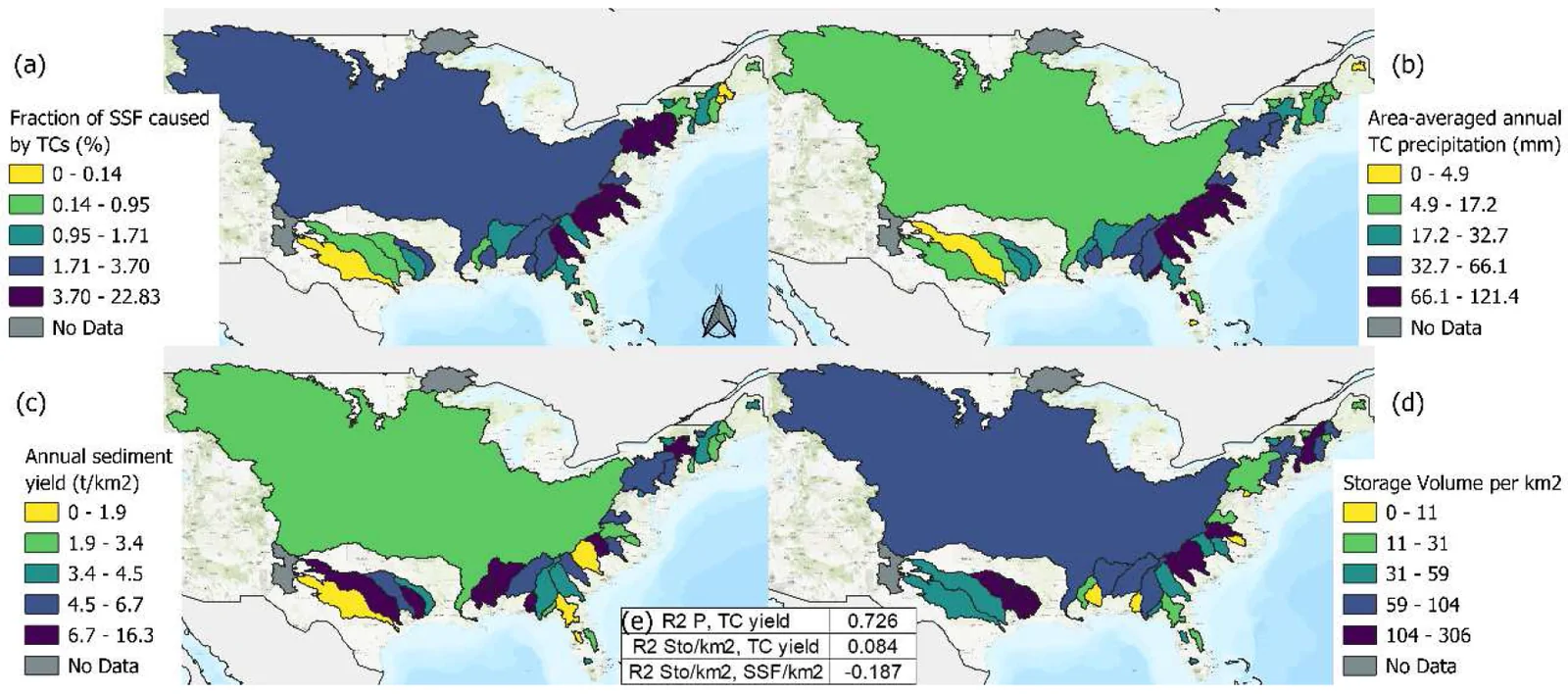
a) Fraction of the total SSF caused by tropical cyclones – an indication of TC precipitation effect magnitude in sediment transport, b) annual tropical cyclone precipitation, area-averaged – indicating the exposure of each basin to TCs, c) Total annual sediment yield of each basin, d) storage volume in acre-feet divided by catchment area (km2) – indicates the level of regulation of a basin, e) correlations between annual TC precipitation (b) and TC yield fraction (a), between storage (d) and TC yield fraction (a), and between storage (d) and total yield (c). A table of the excess mass of sediment transported by TCs in each station and its representation relative to the total mass of sediment transported in the river is presented on the SI (Tab. S01). Barriers dataset: national anthropogenic barrier dataset (NABD)^22^.

TCs resulted in a combined excess sediment mass transported of 278,489 tons of sediment per year in the eastern United States, compared to 12.15 million tons of total sediment mass in the area annually (Tab. S01). Excess sediment is defined as the sediment transported directly due to quickflow (i.e baseflow-separated discharge) within the sediment transport window for each TC in each location. The excess sediment mass transported through each basin varies in both absolute value (0 to 163,000 tons annually) and relative representation of the total mass of sediment transported in a year (0 to 22.8% annually) (Tab. S01) which can be visually observed in Fig. 1.

Precipitation is deeply connected to sediment flux. The correlation between TC precipitation and TC yield is strong. However, there is a weak positive correlation (0.084) between storage volume and TC yield, and negative correlation (−0.187) between storage and sediment flux from all precipitation types (Fig. 1e). We observe that some of the TC events generate sediment responses in every basin, while others do not (Fig. S04), which can be connected to the areas affected by the TC precipitation for each event.

### Responses to TC and non-TC precipitation

**Figure 2 –:**
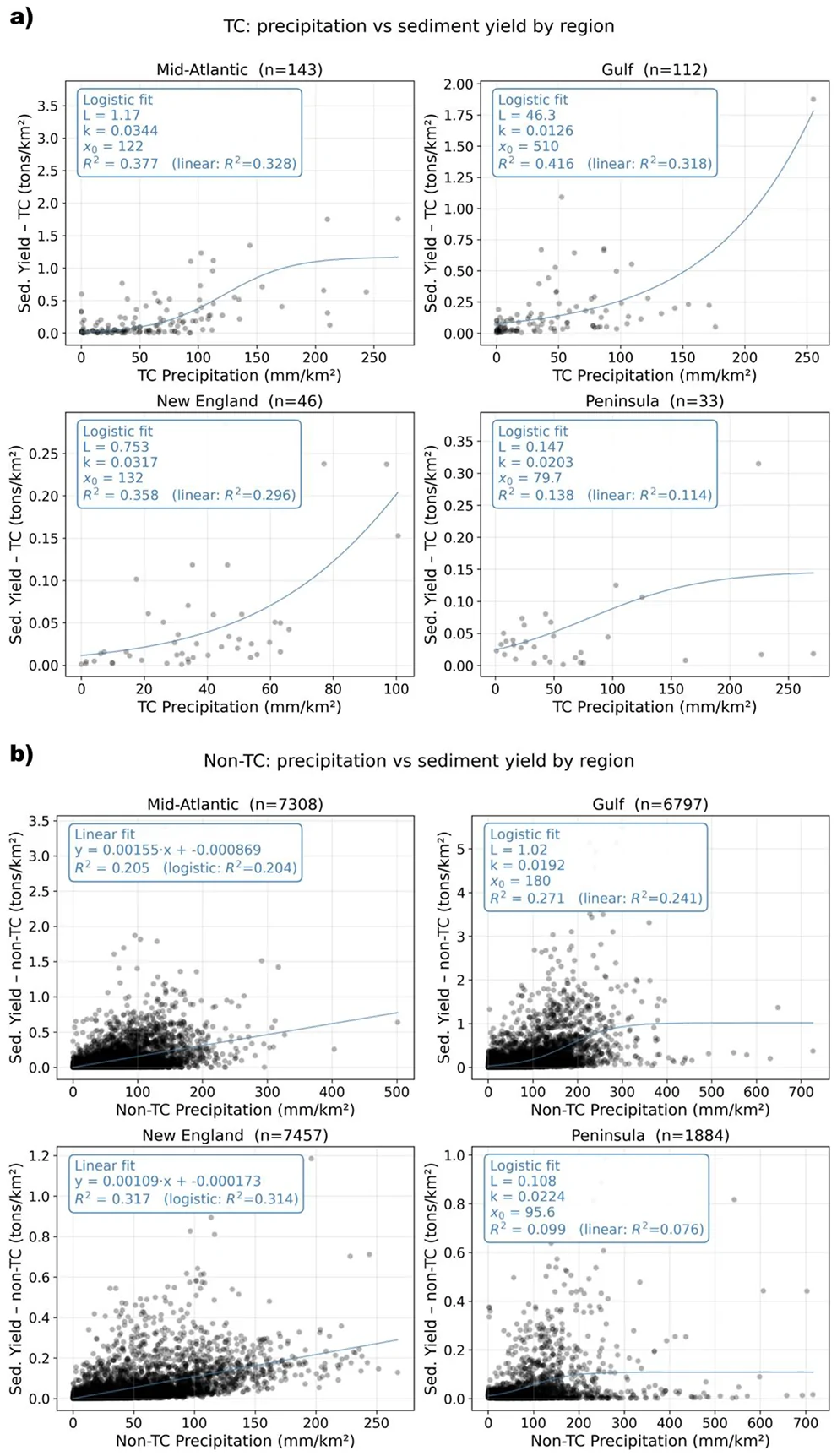
Connections between TC precipitation and sediment transport in rivers. a) relationship between tropical cyclone precipitation and excess sediment yield. Each point represents the sediment transport response of one tropical cyclone in one basin. b) relationship between precipitation unrelated to tropical cyclone, and excess sediment yield. Each point represents the sediment transport response of one precipitation event in one basin. Precipitation events are defined as wet periods separated by at least 24h of dry periods. Reproduced for total (baseflow and quickflow) yield: Fig. S05. Reproduced only for observed events: Fig. S06.

TC events show distinct sediment response shapes compared to non-TCs across all regional clusters (Fig. 2). The fitted curves on Fig. 2 assist interpretation and are not to be taken as models. Non-TC events have higher spread. TC-driven sediment flux has a distinct response pattern, while non-TC events of similar magnitude and return interval have less cohesion in their response, suggesting that TC driven flux is different from non-TC flux. Across events of many sizes, event-scale TC excess sediment yield (flux per area) rapidly increases with increasing TC precipitation up to a certain accumulated precipitation threshold. The existence of a maximum sediment transport capacity had been observed for TC events in a catchment in China^10^. For non-TC precipitation events, sediment yield either increases linearly or is self-limited, hitting a plateau, but the variance makes linear and sigmoid fits less reliable for non-TC events.

**Figure 3–:**
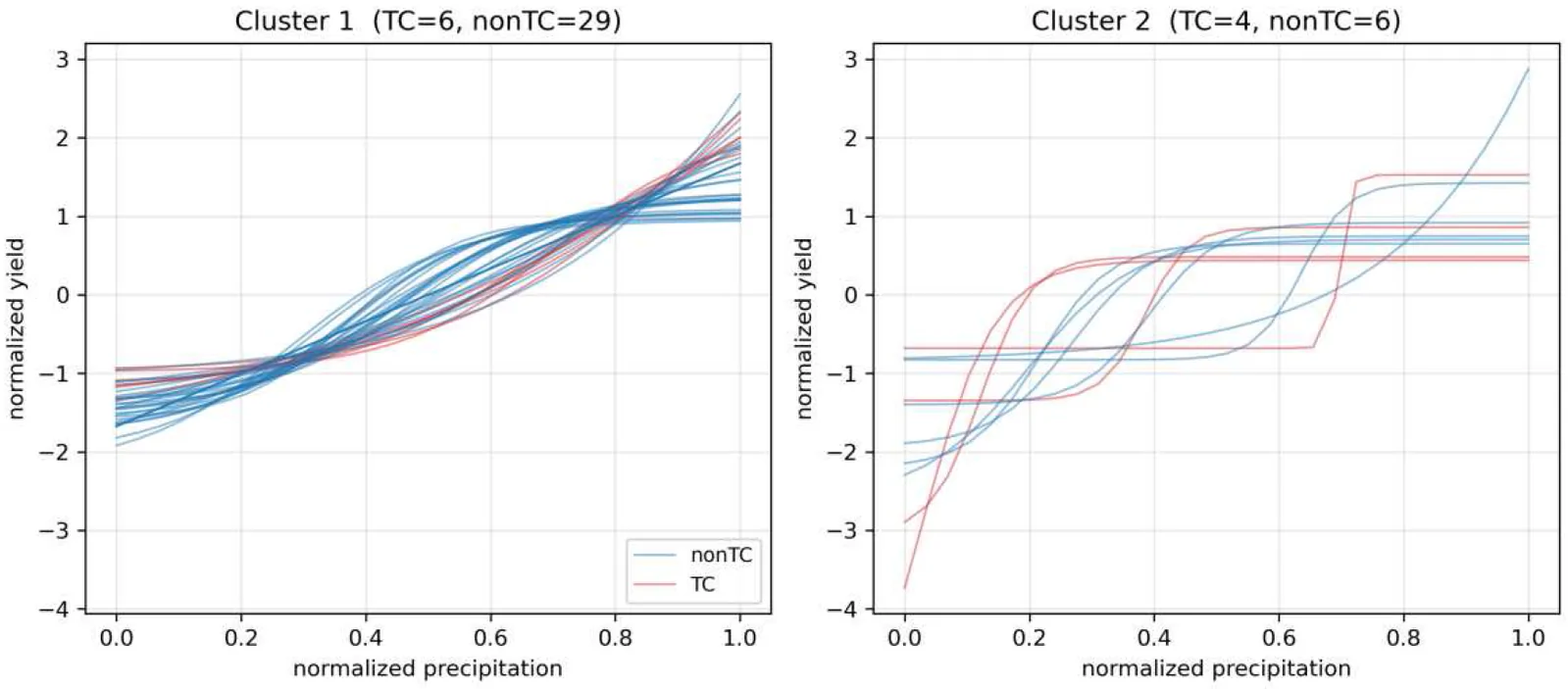
Sediment response shapes for each station for TC and non-TC precipitation, clustered in two groups. Reproduced in total yield: Fig. S07. Reproduced only for observed events: Fig. S08.

**Figure 4 –:**
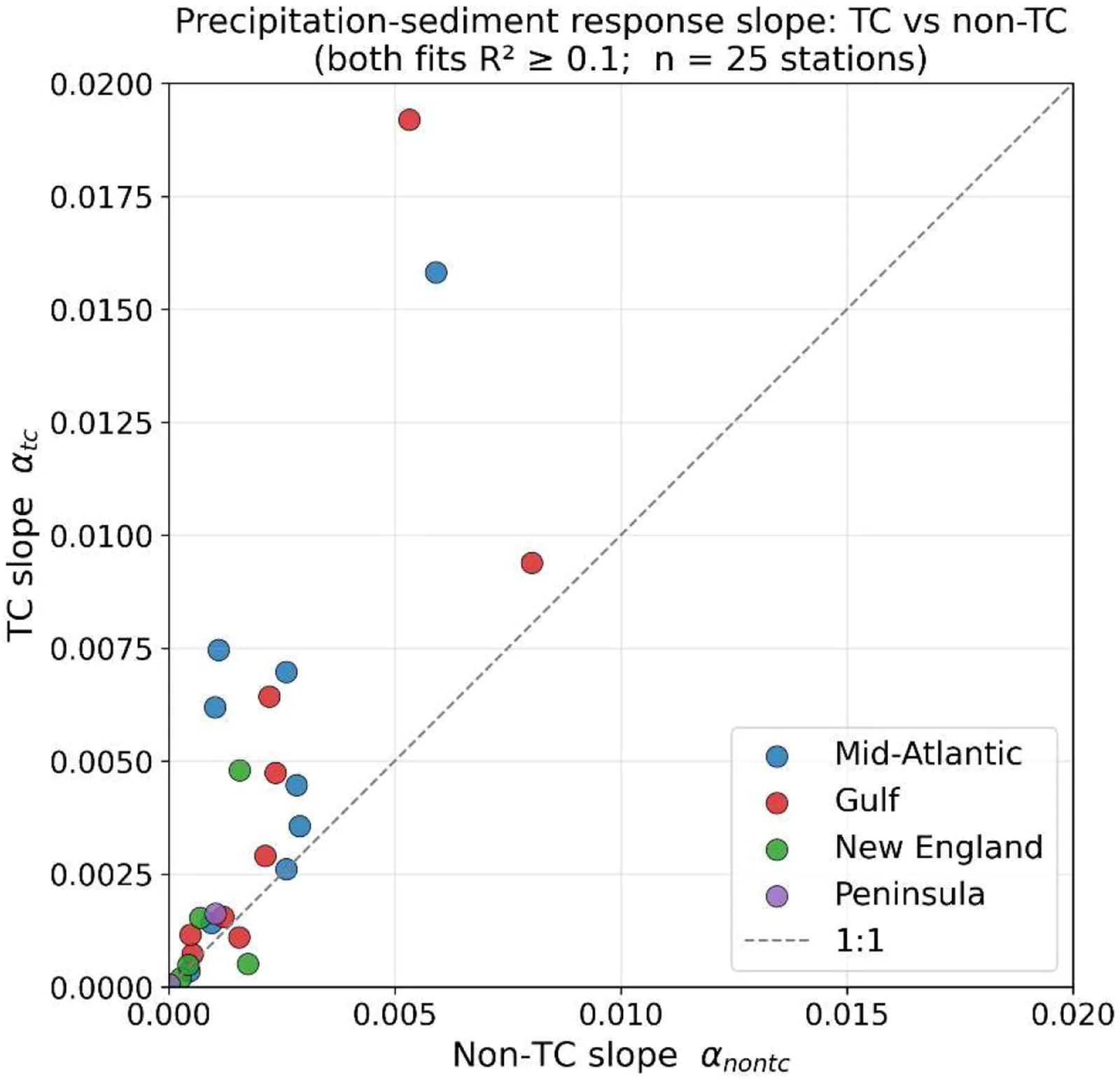
alpha parameter (slopes) for linear approximations of the relationships between event precipitation and sediment response for TC and non-TC events. Only basins which had linear correlations over 0.1 for both, considered meaningful, are presented in this figure. Reproduced in total yield: Fig. S09. Reproduced only for observed events: Fig. S10.

The shape of the response to TC and non-TC precipitation appears to be different, which can be observed by nondimensionalizing the response curves and separating them into two self-supervised clusters (Fig. 3). The biggest differences are observed in Shape Cluster 1. Non-TC precipitation tends to plateau the normalized yield curve, while TC precipitation has increasingly more yield as precipitation increases. We recognize that the sediment response curves are limited to the data we have, and differences in response curves may be skewed by extreme TCs that occurred during our timeline. However, if those curves reflect extremes for both TC and non-TC, then river sediment responses to TC precipitation are fundamentally different from those to non-TC precipitation. In most locations, TC-induced sediment flux responses can increase indefinitely with precipitation totals, or the inflection point occurs with more precipitation, i.e., the rapid increase in sediment flux will be achieved after higher precipitation totals. This does not mean that TC precipitation has less hazard potential, since the sediment totals for TC precipitation tend to be higher for the same amount of non-TC precipitation (Fig. 4). For total yield and filtering just events with observations, the sediment response shape is more scattered, not showing clear signatures of TC and non-TC (Fig. S07, Fig. S08).

If only linear approximations of the relationship between event precipitation and sediment response are considered, we can compare the slope (alpha parameter) that indicates the marginal contribution of each precipitation unit to riverine sediment response (Fig. 4). Most of the stations are above the 1:1 line, indicating that TC precipitation has stronger effects on riverine sediment transport than non-TC precipitation, for the same volume of precipitation. For non-baseflow separated sediment yield and filtering events with at least one SSC observation (Fig. S09, Fig. S10) the same effect is observed, showing the signal is strong.

### Unit response

**Figure 5 –:**
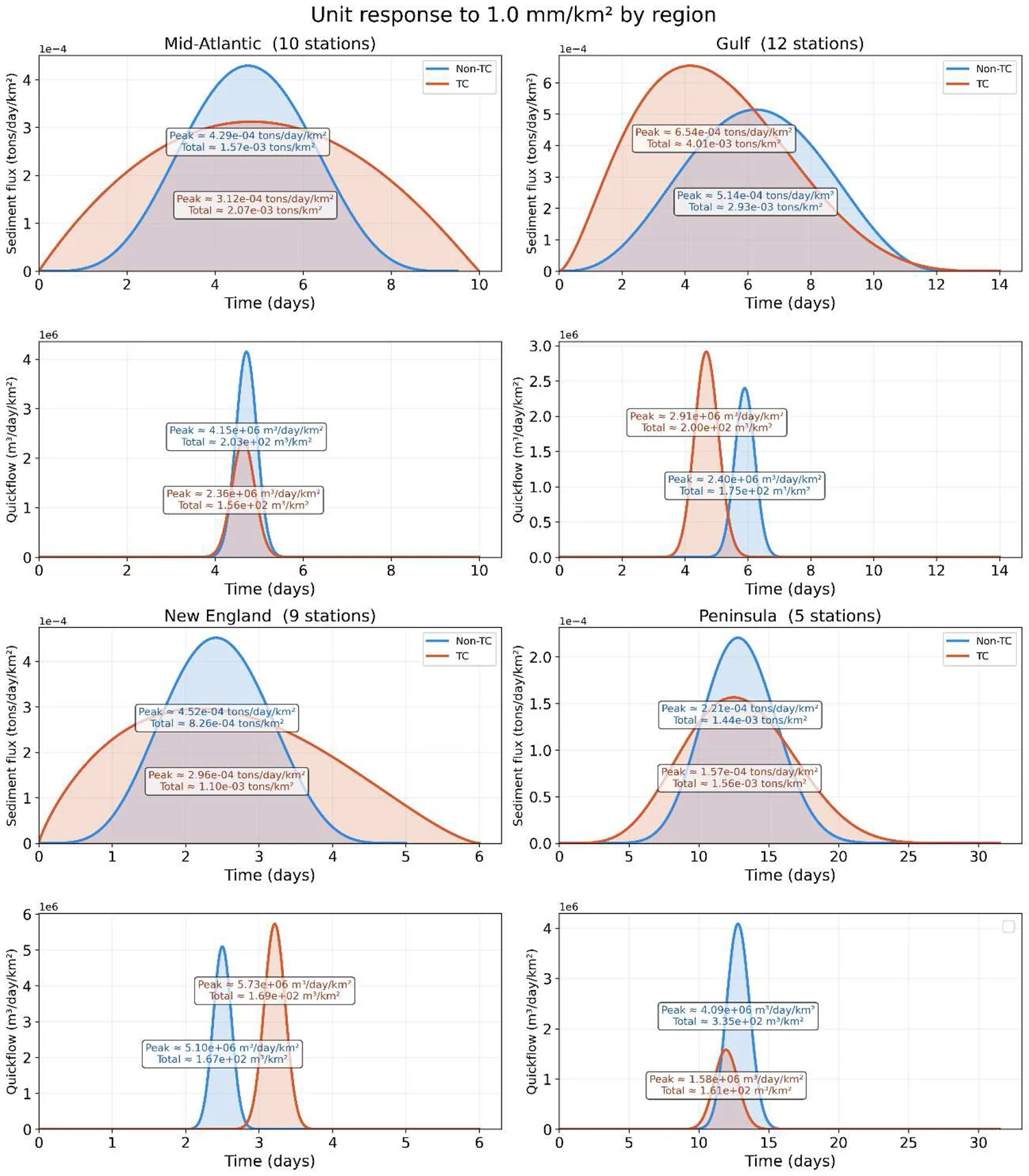
Unit responses to 1 mm/km2 of TC and non-TC precipitation, for excess sediment flux and quickflow (baseflow-separated discharge), aggregated by clusters. Reproduced in total yield: Fig. S11. Reproduced only for observed events: Fig. S12.

TC-associated 1mm of precipitation is more impactful than the same precipitation volume for non-TC events (Fig. 5). The unit responses for quickflow (excess discharge) are more transient than the excess sediment responses, for all geographical clustered areas. Sediment responses were larger in total mass for the TC events, for all geographical clusters, even those in which quickflow responses were lower (Mid-Atlantic and Peninsula). Only in the Gulf area the TC event sediment response also had a higher peak of sediment transport, showing that for TC precipitation, responses are more spread out in time but result in larger mobilized volumes. The peak of sediment transport by TC events happens earlier than for non-TC events in all areas. For quickflow, in the New England region, the discharge peak due to non-TC precipitation occurs, on average, earlier; in all other clusters, it occurs later. For sediment yield calculated including baseflow (Fig. S11), the sediment response shapes are more rectangular for both TC and non-TC, indicating that for the average event the base flux of sediment is large in comparison to the excess sediment flux. If using only events with SSC observations (Fig. S12), the plots are biased toward long-duration TC events (which have higher probability of having a remotely sensed observation).

## Discussion

In this work, we present a remotely sensed estimate of the river sediment transport response caused by tropical cyclones in eastern US rivers, and an estimate of their relative importance to the total sediment yield of each river basin, as well as compared the quickflow and excess sediment responses to non-TC precipitation events in the same areas.

Our results suggest that, in general, excess sediment load in rivers is more strongly linked to TC precipitation than to precipitation from non-TCs in the Eastern United States, and that for similar amounts of precipitation, TC precipitation is more impactful in terms of sediment load, agreeing with findings from other published work^1,3^. TC precipitation tends to generate higher integrated river sediment responses, longer but lower-peaked unit responses, than non-TC precipitation. Therefore, the need to study TC precipitation specifically and separately from other precipitation sources is reaffirmed.

The Gulf area shows both higher peaks and total transported mass for TC than non-TC. This could be due to the trajectory that TCs have relative to the river network orientation^1^ that may cause “resonance” as flood and sediment waves from different parts of the watershed arrive at the outlet at the same time. Precipitation spatial moving direction matters as it has been suggested that TCs are increasingly tracking inland and towards the Northeastern river basins and other basins that historically experience less TCs. As a result, these regions are increasingly susceptible to TC-driven extreme sediment fluxes and their downstream impacts (Fig. S01). In addition, it has been shown that precipitation intensities associated with TCs have increased^23^, which can increase quickflow and sediment flux in rivers. As an example of recent impacts of TCs, in 2024 Hurricane Helene caused landslides and widespread flooding in western North Carolina and eastern Tennessee^13^, directly causing 219 deaths and an estimated damage of US$ 78.7 billion^24^.

Riverine sediment transport in response to TCs reacts more strongly to an increase in precipitation over a certain precipitation threshold. One hypothesis that could explain this observation is cascading geomorphic events triggered by TCs. Examples of cascading events include landslides, mass wasting, and riverbank failure that tend to occur abruptly and rapidly and are caused by extreme rainfall (in both duration and intensity). For example, those were recorded for Helene in the eastern US^4^ and for Maria in Puerto Rico^25^. Landslide debris carried by post-TC streamflow were identified as river sediment flux drivers in Taiwan^1,26^. The mobilization of mass wasting sediment and consequent abrupt increase in sediment concentration in the affected water bodies could explain the slope change in sediment responses. The estimated inflection point of the Mid-Atlantic cluster is 122 mm of accumulated precipitation, which is within the range of total accumulated precipitation for debris flow triggering identified for the Blue Ridge in Virginia (estimated between 116 and 360 mm depending on rainfall duration and intensity)^27^.

The fraction of excess SSF attributed to TC precipitation is more linked to watershed exposure to tropical cyclone precipitation than the degree of streamflow regulation (Fig. 1e). The effect of storage volume on sediment flux at the outlet is negative, as expected, during normal conditions, but slightly positive during TC-related sediment peaks. This may indicate that dams and reservoirs are less efficient at trapping sediment during extreme precipitation. Alternatively, this could be caused by opening dam gates or releasing discharge pulses to manage reservoir water level, run-of-river dam overtopping during high flows, and generally elevated and well-mixed SSC during extremes^28^. Extrapolating from our findings, even high degrees of streamflow regulation already in place may not prevent high sediment transport load peaks. Sediment load peaks are critical to supplying coastal areas with sediment but can also harm ecosystems, damage river adjacent infrastructure, and make drinking water treatment more costly^7^.

Our work has known limitations, that guide the scope of our interpretations. It is not possible to ensure that mechanistic processes involved in sediment transport during extreme events caused by TC are similar to those during regular (baseflow) times or during extremes caused by other mechanisms. In addition, many TC events generated small amounts of precipitation within the watersheds, similar to those generated by precipitation from other processes such as mesoscale precipitation. For large-scale extreme events, additional cascading impacts such as vegetation uprooting and bridge blocking, causing flooding and alluvial-like sediment deposits can interfere in basin sediment connectivity. In such cases, the relationships between discharge and sediment concentration may be non-linear during high-intensity extreme events. Those processes were not studied within the scope of this manuscript.

## Concluding Remarks

We present the first analysis focusing on sediment transport response of TCs for the entire eastern US, highlighting the importance of TCs for riverine sediment transport. Our results point to the cascading hazards potential of inland TC track migration and increases in precipitation intensity as exposure to TCs could be more important than reservoir presence for SSF responses. This can inform hazard mitigation strategies for controlling excessive sediment flux and mitigating TC water quality effects specifically for TC events. We also highlight that TC extreme precipitation and riverine sediment response are deeply related in non-linear ways, indicating that higher volumes of TC precipitation can have a cascading effect on the mass of transported sediment.

## Methods

### Spatial-temporal precipitation footprints

River sediment flux is influenced by precipitation across the entire watershed. To accurately measure individual TC and non-TC precipitation events, we used spatially-explicit, high temporal resolution precipitation data that links observations to specific events and their type and combined precipitation with the International Best Track Archive for Climate Stewardship (IBTrACS)^29^, a dataset that contains unified information on storm location and corresponding climatology every six hours. The Extreme Precipitation Event Catalogue (EPEC)^30^ is a novel database based on the Analysis of Record for Calibration (AORC)^31^. AORC is the approved precipitation database for the National Water Model and is regularly applied for the calibration and validation of hydrologic, hydraulic, and hydrodynamic models. TC precipitation is quantified using the Covariance-informed spatiotemporal clustering (CISC) method^32^. This method combines space time separable covariance modeling^33^ with spatiotemporal clustering^34^ to define space time footprints representing discrete events in geophysical data. TCs are well represented in this framework for two reasons, 1) AORC is a high-resolution observational database that is relatively unbiased compared to modeled or reanalysis datasets and 2) precipitation intensities associated with tropical cyclones will yield relatively more threshold exceedance counts than for other storm types, indicating the superior quality of tropical cyclone event extents and durations within our database.

Combining the EPEC data with IBTrACS and the watershed boundaries, we can compute watershed-scale precipitation for all events across all watersheds, and how the same TC event impacts multiple watersheds along its track. We have the precipitation totals (mm) individualized for each catchment associated with a station and for each TC, as well as peak values and peak times for intensity (mm/h). For non-TC events (total: 23,446 event-station pairs), which can be small scale and highly ephemeral, the event attributions were given intra-basin only (non-TC events were not connected across basins). Non-TC events were defined as wet periods (at least 0.5 mm of precipitation) separated by at least 24h of dry periods. We analyze all TCs that occurred from 2013 to 2024 resulting in a total of 33 events with valid data (334 event-station pairs).

### SSC and Sediment flux

We estimated suspended sediment concentration (SSC) at USGS discharge gauging sites using the SSC module of the Surface Water and Ocean Topography (SWOT) Confluence software^20^, based on Harmonized Landsat Sentinel-2 (HLS) imagery^35^, and with a recurrence time of 1.4 days in average without considering cloud coverage. The module predicts SSC, using a mixture-of-experts of neural networks, to a mean absolute error of 59.34 mg/L and median error of 13.00 mg/L. In our model, underprediction errors are more prevalent than overprediction errors, and the model has a slight underprediction bias, observed in the signed median error, which is −3.38 mg/L.

Cloud coverage is a known issue that prevents, in some cases, collection of SSC data from satellite imagery after the occurrence of TCs^19^. We experimented with two approaches to mitigate the effects of cloud coverage over our remotely sensed measurements of sediment flux and yield: 1. Selecting and analyzing only TC events and stations with data within the window of time in which the sediment transport response to the TC precipitation is expected to happen, and 2. Regressions connecting discharge measurements and SSC are developed for each area, and the SSC responses are interpolated based on the regression, this way, allowing for every TC to be analyzed. Both choices carry important limitations and the estimate of the response to the TC events is compromised in different ways: for method 1, the cloud coverage biases do not allow for a clear observation of the patterns of the sediment response to TC rainfall, and for method 2, the relationship between Q and SSC is assumed to remain constant in time, which is a simplification. In this manuscript, we present results relative to method 2, and have equivalent analyses for method 1 on the SI.

To estimate suspended sediment flux (SSF, kg/s), we combined the SSC estimates with daily discharge measurements from the most downstream, non-tidal USGS gauge across representing 40 different watersheds (Fig. S02). To account for differences in watershed size, we normalized flux by watershed area to calculate sediment yields (kg/(s*km2)) .

Here we focus on the “excess” or baseflow-separated SSF instead of total SSF to characterize the response of the basins to each precipitation event despite the initial flow conditions that can vary across the year. This is especially important when comparing TC with non-TC precipitation events, because TCs occur in a limited season of the year. Excess SSF is calculated by combining the fraction of discharge not attributed to baseflow, using the classical Hysep method^36^, and SSC during the event window. We focus on the excess SSF results, simply referred to as SSF, and have provided results using total event SSF in the SI for comparison.

**Figure 6 –:**
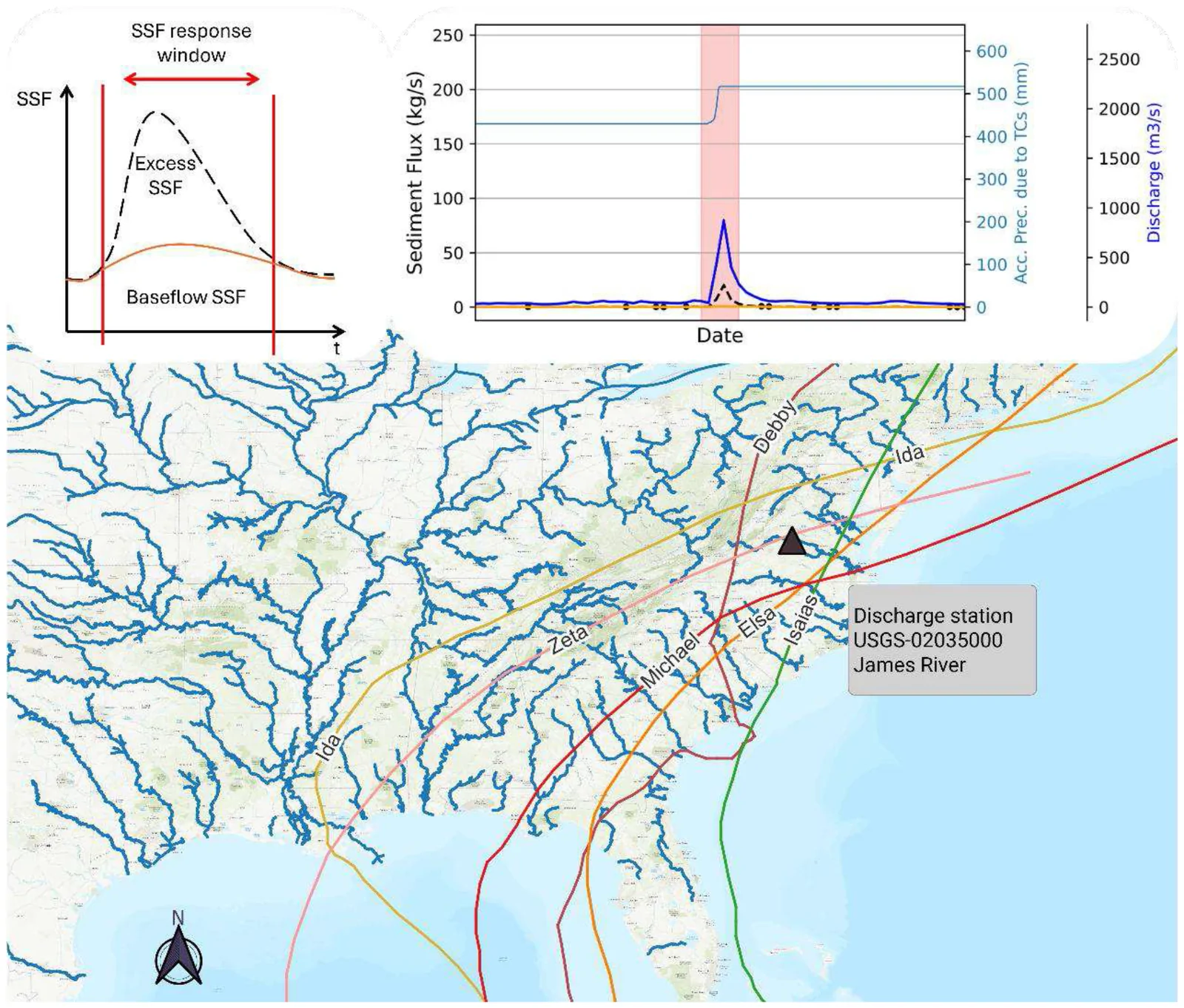
Schema of the integration process of excess sediment flux caused by tropical cyclone precipitation. TC tracks in the map: IBTrACS^39^.

To link sediment flux to precipitation events identified by the EPEC database^30^, we defined an event occurrence window based on the recorded start and end times of precipitation within the watershed as well as the temporal correlation between total SSF and precipitation. First, we use the daily time series of total SSF and precipitation to calculate the lag (in days) for which precipitation is most highly correlated with total SSF for each of the stations. The occurrence window of sediment response to a precipitation event then starts in X+lag in which X is the start date of precipitation and ends in Y+lag in which Y is the end date of the precipitation. Excess sediment yield – integrated excess sediment flux per area unit within the occurrence window - is attributed to the precipitation event (Fig. 6).

### Data Analysis

To identify the effects of TC and non-TCs on sediment response, we examined precipitation-sediment flux relationships at the scale of individual events and across many events as well as at site specific and regional spatial scales. First, to isolate the sediment response shape, we fitted linear and sigmoid curves between TC and non-TC precipitation and sediment flux events at each. The best fit in terms of higher R2 was identified for each station. For the stations with sigmoid approximation, we further characterized the sediment response shape by removing the effect of magnitude by nondimensionalizing based on z-scores of x and y axes. The curves were then grouped using K-means clustering into two to five shape clusters showing the characteristic sediment response curves for TC and non-TC events.

To overcome data limitations at individual stations and identify sediment response at the regional scale, we also divided the stations into 4 geographic clusters (reference fig. S03 for their locations) to derive regional sediment response curves and unit responses and observe inter-region response differences. The geographic clusters' locations were selected based on their spatial location in terms of coast and latitude.

At the event scale, we used a fundamental method of the hydrologic “unit” response to understand similarities and differences between the response of TC and non-TC river network response. The unit hydrographs and hydro-sediment graphs show how one mm of precipitation caused by TCs and non-TCs affects the discharge and sediment transport responses in rivers. This is done by isolating the response to each event and calculating important statistics that help guide the shape: the peak, the day of the peak, and the duration of the response. The responses are aggregated by geographic cluster, but individual plots for each station can be found on the SI.

Lastly, sediment connectivity, or the efficiency of water-based sediment transport from the entire watershed to the basin outlet, is highly impacted by reservoir storage and the nature of sporadic downstream remobilization of sediment over various timescales ^37^. Sediment connectivity and storage is well studied^6^ and not our focus, however we used the national anthropogenic barrier dataset (NABD)^22^ to estimate watershed scale metrics for reservoir regulation, such as total reservoir storage volume, to provide additional context to interpret sediment response to TC across the 40 watersheds.

### Limitations

We focus on suspended sediment flux because it makes up a majority of total flux in large rivers^38^ and is measurable across many watersheds. Further, we recognize remote sensing only captures surface SSC and can underestimate high concentrations^18^. Here we are focused on comparing our flux estimates across events and watersheds and this bias does not impact our goals of analyzing the sediment transport responses to TC precipitation. Our goal is not to generate the most accurate flux estimates, but comparable response curves over space and time to understand the role of TC versus non-TC precipitation in sediment transport.

### Validation of sediment mass totals

Total annual sediment transport figures are within the same order of magnitude as sediment load estimated by other sources. For the Hudson River, the annual average mass estimated by our model was 143,956 tons. An adjustment factor of 4.454 has been proposed to convert satellite-derived SSF to field estimate depth-integrated sediment flux^40^. If applied to our Hudson River result, 641,180 tons. A previous Acoustic Doppler Current Profiler (ADCP)-based work estimated the annual average between 2002 and 2006 at 736,250 tons^41^, which is consistent with our estimate. For the Mississippi River, the annual total estimated by our model is 7.59 million tons, if the factor is applied to our result for the Mississippi River, it results in approximately 33.81 million tons. A previous work had estimated between 81 and 103 million tons based on in-situ measurements made in 2008 to 2010 in the Mississippi River near Baton Rouge, Louisiana, USA^42^. Similarly, for the Susquehanna River, we estimated 0.396 million tons, with the factor, 1.76 million, lower but close to the figure independently estimated by USGS of 2.2 million annually between 1991 and 2012^43^.

### Excess sediment flux methodology validation

Our analyses conducted using other methodologies reiterate the need for baseflow separation of discharge, and for interpolations for TC events without remote sensing observations. Other recent publications have also employed baseflow separation for suspended sediment analyses^44^. Precipitation for both TC and non-TC events generates scattered and inconsistent responses in non-baseflow separated total event yield (Fig. S05), contrasting with the clear effects shown for baseflow-separated SSF, presented in the main text (Fig. 2). Likely, the presence of small magnitude events that do not directly generate significant sediment responses but occur in high-baseflow periods confounds the analysis and reinforces the need for baseflow separation for correct event SSF attribution. For excess sediment yield (flux by area) only calculated for events with one or more observations (Fig. S06), TC-SSF relationships are similar in shape to when all events are accounted for, and non-TC events SSF responses are more scattered than for the interpolated time series.

## Supplementary Material

This is a list of supplementary files associated with this preprint. Click to download.

• SI.pdf

## Data Availability

Precipitation data from EPEC is fully available^30^ at https://doi.org/10.17603/DS2-22CE-N609. IBTrACS is made available by NOAA^39^ at https://www.ncei.noaa.gov/products/international-best-track-archive. Discharge data was pulled from USGS’s Water Data for the Nation portal (https://waterdata.usgs.gov/). HLS optical imagery is made available by NASA^35^ and accessed through download links acquired from the Common Metadata Repository (CMR) (https://www.earthdata.nasa.gov/engage/open-data-services-software/earthdata-developer-portal/cmr-api). NABD is provided by USGS^22^ at https://water.usgs.gov/catalog/datasets/922683e5-7188-41e0-8e71-e38219731d37. The SSC modeled by our model for each station can be found at DOI: zenodo.21382796 (to be published upon acceptance, peer review link was made available to the editor on cover letter).

## Code availability

The code used to estimate SSF is stored at https://github.com/SWOT-Confluence/ssc_model_deployment (DOI: https://doi.org/10.5281/zenodo.19186575), Version 3.0.0, and the code used to process the TC precipitation and its effect on SSF can be found at https://github.com/luisalucchese/TC-SSF-processing (code to be added publicly at the next review stage).
